# Commentary to the articles of M. Stier (Normative preconditions for the assessment of mental disorder) and T. Schramme (On the autonomy of the concept of disease in psychiatry)

**DOI:** 10.3389/fpsyg.2014.00112

**Published:** 2014-02-20

**Authors:** Gerald Ulrich

**Affiliations:** Klinik für Psychiatrie und Psychotherapie, University Medicine-Charité, Universitätsmedizin BerlinBerlin, Germany

**Keywords:** biological or somatic psychiatry?, biologism or somatologism?, materialistic monism vs. epistemic dualism (or dualism of aspects)?, nominalistic definitions, biological natural science and humane discipline

Both authors agree that:

“*Mental illness is not reducible to brain illness, even when mental phenomena have their basis in the brain*” (Schramme, 2013, p. 8) and

“ …*because of the normative nature of psychiatry, mental disorder cannot be completely reduced to neuronal or molecular processes. […] A mental state as such may well be reducible to the brain, but determining whether this very state is (part of) a disorder or not, is nothing the brain sciences can do*.” (Stier, 2013, p. 8).

Therewith the authors deny the tacit assumption according to which disease in its proper sense can only be somatical but never psychic. This posit, which was propagated under the common term of “*Somatosepostulat*” by the German psychiatrist Kurt Schneider (Schneider, 1950) who dominated the post-war psychiatry. Strangely enough the content of Schneider's posit was later on ascribed to a falsely abridged citation of Griesinger according to which mental diseases were nothing but brain diseases (Griesinger, 1861). In fact he stressed in addition that mental states (“*Seelenzustände*”) may not be reduced to functional disturbances of the brain (“*Funktionszuständen des Gehirns*”).

Definitely no one who is familiar with the second edition of Griesinger's textbook would call this distinguished author to witness psychiatry as a special kind of neurology. Moreover, if a mental disease is nothing but a neurological or a somatic disease respectively, it was logical mandatory to speak of “*Somatic Psychiatry.*” Therefore it is disconcerting that this term is absolutely unfamiliar. Instead, Schneider's posit goes under the disguise of the semantically inappropriate term of “*Biological Psychiatry.*” Following this rational way of thought, the unanswered question arises what actually could be intended by the title of the reviewed symposium “*Biologism within Psychiatry?*” (*Biologismus in der Psychiatrie?)* whereas—strictly speaking—one ought to use the unusual or even inexistent term of “Somatologism.”

By the way, to the present author whose duty consisted only to comment on a freely chosen article (out of six) it is cloudy that neither in the workshop nor in any one of the later on prepared articles an answer was searched for the core issue contained in the workshop-title whether psychiatry suffers from biologism (or not). This seemed to be avoided like a hot potato since it would have implied a debate on the outstanding semantics of “biological,” biologistical or “somatical.”

Schramme states, that the recent publication of DSM V should give occasion for the underlying philosophical aspects of the language of mental disorder to make itself clear within the psychiatric trade. He criticizes DSM IV for using the term “*mental disorders*”:

“*Mental implies a Cartesian view of the mind-body problem that minds and brains are separable and entirely distinct realms, an approach that is inconsistent with modern philosophical and neuroscientific views*.” (Schramme, 2013, p. 8).

One cannot but agree that the progress of psychiatry depends on a logical and semantical consistent terminology. But it is just as disputable that this aim can be reached by simply eliminating the colloquial term “*mental*” being used as a synonym of “*psychic*” and/or an antonym of “*somatic.*” Why should *the concept of mental illness* be *autonomous from somatic medicine*, as Schramme claims?

The real problem to be solved is not an outdated Cartesian view of substance dualism, being scarcely advocated by any of the contemporary psychiatrists, but the prevailing materialistic monism or eliminative reductionism (e.g., Paul and Patricia Churchland, Armstrong, Quine Ryle, Skinner, Crick etc.) which is being camouflaged by the term “biology/biological” being ill-posed because opposite to the sense intended.

There is only one epistemological solution which goes back to Spinoza (1890). Among the contemporary exponents of this solution the best known is Habermas (Habermas, 2004) who prefers to speaks of “*Epistemic dualism.*” Another notion of this concept which dispels the myth of the unsolvable mind-brain problem is that of “*Aspektdualismus*” (Ulrich, 1990, 1997, 2006a,b, 2013).

About a decade ago a German psychiatric chair holder wrote about his vision of an integrated clinical-neuroscientific field with psychiatry as a special focus but no longer existing as an autonomous field (Maier, 2002). The Psychiatrist was redefined as a Clinical Neuroscientist or Clinical Psychopharmacologist.

Recently the director of a renowned Max-Planck-Institute for Psychiatry confessed in an interview that he surely was a better chemist than psychiatrist (Holsboer, 2005). By an editorial, entitled: “*Are we still in need of psychiatry as a special field within medicine?*” Ulrich (Ulrich, 2006a) demanded that psychiatrists should discourage any attempt to abandon psychiatry as a distinct discipline. He referred to the demand of *Aspect Dualism* being valid for medicine as a whole. Accordingly, “either-or interrogations” have to be replaced by “as-well-as” ones. Thus, it was undue to beg the question whether a hysteria is a brain disease or a psychological disease, or whether a depressive disorder is a biochemical or a psycho-social disorder. Such nominalistic definitions are equally misguided as the question whether an altarpiece should be labelled as an antique or a sacred object. By a recent monograph Ulrich defined Psychiatry both as a Biological Natural Science *and* a Humane Discipline (Ulrich, 2013).
